# Reference Standards to Support Quality of Synthetic Peptide Therapeutics

**DOI:** 10.1007/s11095-023-03493-1

**Published:** 2023-03-22

**Authors:** Diane McCarthy, Ying Han, Kevin Carrick, Dale Schmidt, Wesley Workman, Paul Matejtschuk, Chinwe Duru, Fouad Atouf

**Affiliations:** 1 Global Biologics, United States Pharmacopeial Convention, 12601 Twinbrook Pkwy, Rockville, MD 20852 USA; 2 Workman Biotech Consultants, LLC, Cottleville, MO 63304 USA; 3 Analytical & Biological Sciences, Medicines & Healthcare products Regulatory Agency, South Mimms, Hertfordshire, EN6 3QG UK

**Keywords:** analytical characterization, content assignment, peptide pharmaceuticals, quality attributes, reference standards, stability testing

## Abstract

**Purpose:**

Peptides are an important class of therapeutics. Their quality is evaluated using a series of analytical tests, many of which depend on well-characterized reference standards to determine identity, purity, and strength.

**Objective:**

Discuss approaches to producing peptide reference standards, including vialing, lyophilization, analytical testing and stability studies.

**Methods:**

Case studies are used to illustrate analytical approaches to characterize reference standards, including methods for value assignment, content uniformity, and identity testing. Methods described include NMR, mass spectrometry, and chromatography techniques for identity testing and HPLC and GC methods for assessing peptide content and impurities.

**Results:**

This report describes the analytical strategy used to establish peptide reference standard and illustrates how results from multiple labs are integrated to assign a value to the final lyophilized vial. A two-step process for value assignment is described, which uses a mass balance approach to assign a quantitative value to a bulk peptide material. The bulk material is then used as a standard to assign a final value to the vialed material. Testing to confirm peptide identity and to ensure consistency of the vialed material is also described. Considerations for addressing variability, identifying outliers, and implementing stability studies are also presented.

**Conclusion:**

The methods and case studies described provide a benchmark for best practices in establishing the preparation, analytical testing, handling, and storage of peptide reference standards for the pharmaceutical industry. Some peptide features, such as chiral or isobaric amino acids, may require additional techniques to ensure a full characterization of the peptide reference standard.

## Introduction

Peptide pharmaceuticals are valuable therapies and will continue to be a source of future therapies. The FDA has differentiated peptides and proteins by defining that a peptide consists of 40 amino acids or less [1]. Peptide pharmaceuticals can be produced synthetically or through recombinant DNA technology and can be considered the “same” regardless of their route of manufacture [2]. The analytical characterization of peptides is similar for both synthetic and recombinant products, but there may be some differences in impurities based on manufacturing process. When assessing the quality of a peptide pharmaceutical, the attributes of greatest importance are (i) primary sequence, (ii) secondary structure, (iii) oligomer/aggregation state, and (iv) impurities and degradation products. In some instances, it may be important to also address tertiary structures [3]. Additionally, pharmacological attributes such as biological activity are important to address.

Numerous regional regulatory authorities have established official testing procedures to ensure that the drug products provided to the public are of acceptable quality. These official testing procedures often require calibration against a higher order physical standard (e.g., pharmacopeial reference standard) with an appropriately assigned value. Such reference standards are essential for assessing the quality attributes of therapeutic products such as peptides, as described in pharmacopeial methodologies and specifications [4, 5]. Since the quality attributes (e.g., assay, purity) of the drug substance should be established against a well characterized reference standard, it is imperative that those attributes remain unchanged in the reference standard and that its original purity determination is valid under the conditions in which it will be used [6]. The reference standard must be protected from degradation, which requires suitable control of storage and shipping after the reference standard has been qualified so that the integrity of a specific lot of reference standard is maintained throughout its lifecycle.

This article describes the type of material and testing strategies for reference standards that support peptide drug products defined in the United States Pharmacopeia and the National Formulary (USP-NF). The United States Pharmacopeia (USP) has published official monographs and general chapters that provide a quality standard for drug products across the world. USP's standards have been recognized in many countries worldwide and have also been integrated into the laws of various countries. These published documentary standards focus on the quality attributes of identity, purity, safety, and potency of the drug products regulated by authorities that enforce these standards [4]. Physical standards, such as the USP peptide reference standards described herein, are procured, tested, and confirmed with assigned values that are used with the USP published methodologies to evaluate the quality of drug products that meet the requirements under the USP documentary standards. The assigned value of a USP physical standard is only relevant when used with the official USP methodology and users may determine the suitability of the USP reference standards when employing another analytical methodology. There are some process-related impurities methods included in USP peptide monographs and general chapters (e.g., trifluoroacetic acid, acetic acid) that may not require the use of reference standards. Biological activity (i.e., potency) assays are described in USP monographs when the peptides have higher order structure (secondary or tertiary) and where a biological assay is needed to ensure the quality of the drug product.

This report focuses on lyophilized peptide standards, since USP has shifted many peptide reference standards from powdered to lyophilized forms to eliminate the need for the user to determine counter-ions and residual moisture prior to use. The purpose of this report is to illustrate the points to consider when developing a peptide reference standard and to describe the characterization and testing of materials that is required to establish them as reference standards. Considerations for how these reference standards are filled in vials, lyophilized, and tested for stability will be also discussed. The article also describes how content of peptide per vial of a reference standard is determined based on the testing and analysis in multiple laboratories.

## Material and Methods

### Preparation of Lyophilized Candidate Peptide Lots

The peptide reference standards discussed herein are listed in Table I, along with some of their characteristics. Table II lists the quality attributes and analytical methodologies commonly used to assess the quality of therapeutic peptides. Materials described in this report were obtained as bulk solid powders. To prepare the candidate peptide lots for lyophilization, peptides were individually solubilized at concentrations between 2 and 5 mg/g in water for irrigation. The solubilized peptide solutions were dispensed in 1-g aliquots into type I amber glass vials (Schott VAD005, Adelphi Tubes, Haywards Heath, UK), lyophilized and sealed with an appropriate stopper (West 14 mm diameter cruciform halobutyl closures, Adelphi Tubes, Haywards Heath, UK). Trial fillings were performed with a Hamilton MLab 510b syringe dispenser (Esslab Ltd, Westcliff on Sea, UK) and candidate batch filling was performed on a AFV 5060 vial filling line (Bausch & Strobel, Ilshofen, Germany). Trial lyophilization cycles were performed with a Virtis Genesis 25EL lyophilizer (Biopharma, Winchester, UK). Once the lyophilization cycle was developed final lyophilization of the individual candidate peptide lots were performed with a CS-150 Serail lyophilizer (Serail, Le Coudray St Germer, France).

**Table I:** Peptides and Their Respective Characteristics

Peptide	Length (# aa)	Cyclic or linear	D-amino acids
Bivalirudin	20	linear	yes
Desmopressin acetate	8	cyclic	yes
Exenatide	39	linear	no
Gonadorelin	10	linear	no
Leuprolide	9	linear	yes
Oxytocin	9	cyclic	no

**Table II:** USP Peptide Quality Attributes and Analytical Methodology

Peptide quality attribute	Test(s) method^1^	Objective
Identity	NMR	Confirm structural features
	LC–MS/MS	Peptide mass and Amino acid sequence
	HPLC	Compare to standard (previous lot or other well characterized material)
	AAA	Distinguish Ile vs. Leu
	Chirality	Confirm D-amino acids
Purity	Chromatographic purity by HPLC	Determine quantity of peptide impurities
	Acetic Acid by HPLC	Determine acetic acid content
	Trifluoroacetic acid by HPLC	Determine TFA content
	Residual solvents	Determine other residual solvents
	Residue on Ignition	Determine inorganic impurities
Strength^2^	HPLC	Determine quantity of therapeutic peptide (dose)

^1^Monographs and general chapters in the *United States Pharmacopeia* and the *National Formulary (USP-NF)*,

^2^Strength is referred to as *Assay* in USP monographs and general chapters

### Physical Testing of the Lyophilized Candidate Peptide Lots

A set of physical tests were performed to evaluate the quality of the filling operation for each of the peptide lots. Mean dry weight of each of the fills and the coefficient of variation were tested to determine the uniformity of the fill. The residual moisture content and headspace oxygen content of each of the peptide fills were also determined. Residual moisture was determined destructively by coulometric Karl Fischer titration (Mitsubishi CA-200, A1-Envirosciences Ltd, Blyth, UK), opening the vials within a dry nitrogen environment to minimize moisture ingress. Oxygen content of the headspace gas was determined non-destructively by frequency modulated spectroscopy (FMS-760, Lighthouse Instruments, Charlottesville, VA, USA) calibrated with bespoke 0% and 20% oxygen standards in sealed containers. Oxygen content in headspace is important to measure when working with peptides that contain oxidizable amino acids such as methionine.

### Analytical Testing of the Lyophilized Candidate Peptide Lots

Testing was performed on the bulk material to confirm identity and establish a mass balance calculation for use in value assignment to the lyophilized vials. The tests listed below indicate which material (bulk, lyophilized vials or both) were analyzed. Different peptides from Table I were selected to illustrate the type of quality attribute described in Table II. The specific monograph RP-HPLC method for each peptide was used to evaluate the peptide product before and after the lyophilization operation.

#### NMR (bulk)

NMR was used as one of several orthogonal methods to establish identity. For the example reported here, 18.856 mg of leuprolide acetate was solubilized in 0.6 ml of Dimethyl Sulfoxide-d6 (+ 0.03% v/v TMS). The proton, carbon, COSY, NOESY, TOCSY, ^1^H/^13^C HSQC, ^1^H/^13^C HMBC, ^1^H/^15^N HSQC, and ^1^H/^15^N HMBC NMR spectra were collected and compared to expected values for each amino acid.

#### Mass Spectrometry (bulk)

Electrospray LC–MS/MS was used to confirm the mass of the peptide and to verify the amino acid sequence. For identification of leuprolide acetate presented here, a 1 mg/ml solution of leuprolide acetate in water was analyzed on a Waters CORETECS UPLC T3 column (150 mm × 2.1 mm, 1.6 μm) using a linear gradient of 0.1% formic acid in water (solution A) to 0.1% formic acid in acetonitrile (solution B) and a flow rate of 0.4 ml/min. A mass range of 100 – 2000 m/z was used for MS mode and a range of 50 – 1300 was used for MS/MS.

#### Chromatography (bulk and lyophilized vials)

RP-HPLC was used to determine the lot homogeneity, stability, identity, content, and purity for the peptide reference standards. For the analysis of leuprolide acetate shown here, 20 μl of leuprolide acetate was injected on a YMC Pack ODS-A C18 column (100 mm × 4.6; 3 μm) as described [6] using a flow rate of 1.5 mL/ min and a column temperature of 25°C. Peptides and impurities were monitored at 220 nm. The RP-HPLC methods for other peptide reference standards have been described [6]. Triplicate injections from a single container were used to determine the main peak content.

#### Amino Acid Analysis (lyophilized vials)

Amino acid analysis was used as an identity method to distinguish between leucine and isoleucine in selected peptides, like bivalirudin, that contain both amino acids. Samples were accurately weighed into ampules and solubilized in 6 N HCL with 4% phenol. After incubating 18 h at 110°C, samples were dried by vacuum to remove residual acid, resolubilized, and filtered using a 0.45 μm filter prior to amino acid analysis [7].

#### Chirality (GC–MS) (lyophilized vials)

Samples were hydrolyzed in 350 μl 6 M DCl in D_2_O under vacuum for 8 h at 110°C. After removal of excess reagent by a steam of nitrogen, the samples were esterified with 250 μl of 15% hydrochloric acid-ethyl alcohol for 40 min at 110°C. After cooling to about 40°C, the reagents were evaporated with a gentle steam of nitrogen at 110°C. The residue was acylated in 250 μl trifluoroacetic anhydride/ trifluoroacetic ethylester (1:2) and heated for 10 min to 130°C. After cooling to room temperature, the excess of reagent was removed by a steam of nitrogen and the residues were dissolved in 250 μl dichloromethane and injected onto a Chirasil‑Val column (20 m × 0.3 mm, 0.3 μm). The carrier gas was hydrogen, injector temperature was 220°C, and split was 24.7 mL/min. The initial column temperature of 65°C was held for 3 min, then ramped at 4°C/min to 135°C, and 15°C/min to 180°C, and then held for 10 min at 180°C. The amino acids were identified via retention time and mass spectra.

#### Water Content (bulk)

Water Content was determined by Karl Fischer titration as described in USP general chapter < *921* > *Water Determination* [6]. Water corrected purity was calculated as $\left[purity\right]\times\left[\left(100.0-\%water\right)\;\div\;100\right]$.

#### Acetic Acid (bulk)

Acetic Acid content was analyzed by HPLC method according to the method described in USP general chapter < *503* > *Acetic Acid in Peptides* [6]. Sample solution was injected on a L1 packing column (4.6 mm × 25 cm; 5 µm) and analyzed by gradient elution with monitored at 210 nm.

#### Trifluoroacetic Acid (bulk)

Trifluoroacetic acid content was evaluated by HPLC using the method in USP general chapter < *503.1* > *Trifluoroacetic Acid (TFA) in Peptides* [6]. 20 µL of sample solution was injected on a L1 packing column (4.6 mm × 25 cm; 5 µm) and analyzed by gradient elution with a flow rate of 1.5 mL/min. Trifluoroacetic acid was monitored at 210 nm.

#### Residual Solvents (bulk)

Residual solvents were analyzed by gas chromatography as described in USP general chapter < *467* > *Residual Solvents*. [6].

#### Inorganic Impurities (bulk)

Inorganic impurities/thermal ash were determined by residue on ignition according to USP general chapter < *281* > *Residual on Ignition* [6] or thermogravimetric analysis per general chapter < *891* > *Thermal Analysis* [6].

### Analysis of Variability and Outliers

For the desmopressin example presented here, outlier detection was assessed via residual analysis. Any standardized residual with an absolute value greater than 3.0 was considered an outlier. No individual values were removed. Analysis of variance (ANOVA) showed that one lab had a statistically significant differences when compared with the means of the other five labs. This lab also did not perform water content testing which warranted the removal of the data from further analysis. The within-laboratory variability also differed by lab so labs with higher variability were down weighted for the value assignment using inverse-variance weighting.

The data were analyzed using SAS’ Proc Mixed (Windows version 9.4). Collaborating laboratory is an assumed random effect to allow for correlation of results within-laboratory. The reported confidence interval for assay does not include any contributions from the uncertainty of the value assigned to previous reference lots. The data were analyzed in the original and log scales. The residuals from the original scale analysis better met the normality assumption and was therefore used for analysis.

### Accelerated Stability Studies (lyophilized vials)

Samples of the Candidate Peptide lots were stored at various temperatures and timepoints that ranged from -70°C to 45°C for up to 48 months. Samples were placed in sealed polythene bags and stored in either polycarbonate boxes or sealed aluminum tins within each controlled temperature station. These stations (Model 1200, LMS Cooled Incubators Ltd, SevenOaks, UK) were placed in a controlled temperature room and continuously monitored for temperature but relative humidity was not monitored or controlled. The -20°C samples were held in similar containers but stored within a controlled temperature -20°C cold room. The -70°C samples were stored in a freezer. Storage was logged and samples removed at recorded dates for analysis.

## Results and Discussion

### Fill Precision, Homogeneity, Residual Moisture, and Oxygen Content

It is critical that the fill weight be consistent between reference standard containers if it has the potential to impact the analytical testing. The precision of the fill is evaluated by determining the mean fill weight post lyophilization (Table III). The variability from the fill adds to the variability of the measurement, therefore limiting the % CV of the fill weight is important. When filling and lyophilizing biological and peptide materials for use as a quantitative standard, a target of less than 0.5% coefficient of variation for fill weight between the containers is desirable to ensure good batch homogeneity. A percentage CV below 0.5% will have minimal impact on the measurement and therefore minimal impact on the ability to meet typical specifications of 95.0–105.0% of the labeled value. RP-HPLC was also used to test homogeneity of each peptide lot using lyophilized samples from early, middle, and late in the filling sequence and comparing this with frozen baseline material from the filling operation that was subsequently held sealed at -70°C. Residual moisture and oxygen content are important quality attributes for lyophilized standards contributing to reference standard stability [8].

**Table III:** Physical Measurements of the Lyophilized Peptide Lots

Peptide	Mean Dry Weight (mg)	Coefficient of Variation for Fill Weight (%CV)	Residual Moisture Content (% w/w)	Percent Oxygen Content (%)
Bivalirudin	4.7	0.20	1.40	0.80
Desmopressin acetate	2.0	0.22	1.64	0.57
Exenatide	2.4	0.20	1.11	0.72
Gonadorelin acetate	2.0	0.25	2.79	1.58
Leuprolide acetate	2.0	0.26	1.40	0.60
Oxytocin	2.0	0.22	1.38	0.77

#### Analytical Testing

Once the lyophilized vials meet pre-specified criteria for the fill finish including stability assessment, accuracy and reproducibility of the fill, USP peptide reference standards are extensively tested and evaluated by multiple independent laboratories. The testing reported here included methods that help ascertain identity and monitor stability for specific peptide reference standards presented as examples. The results from the multi-laboratory studies are analyzed statistically to allow assignment of a label value in mg per vial for each of the peptide reference preparations. The label value becomes the official value assignment for each preparation and allows it to be used as an officially designated Reference Standard for the analysis of drug substance and drug product. Descriptions of the test results used to classify a reference preparation as a reference standard are presented in this article using relevant peptides as case studies.

#### Identification

Identification tests are specific for a peptide and, as stated in ICH Q6A [9], “should optimally be able to discriminate between compounds of closely related structure which are likely to be present”. Multiple methods are used to confirm the identity of peptides sequences, depending on the size and structure of the peptides, and the use of these methodologies continues to evolve, and the preferred methods can vary depending on the characteristics of the specific peptide. For example, while NMR has been proposed as an alternative to amino acid analysis (AAA), the use of NMR with larger peptides can yield spectra that are less well resolved [10]. Therefore, multiple orthogonal techniques are typically used to verify the chemical structure and chemical identity of a peptide RS, including HPLC retention time, NMR, MS, and chiral testing. USP Leuprolide Acetate RS is used as an example to illustrate the identification testing that USP conducted during the peptide RS development.

#### Identity by Nuclear Magnetic Resonance (NMR) Spectroscopy

**Fig. 1:**
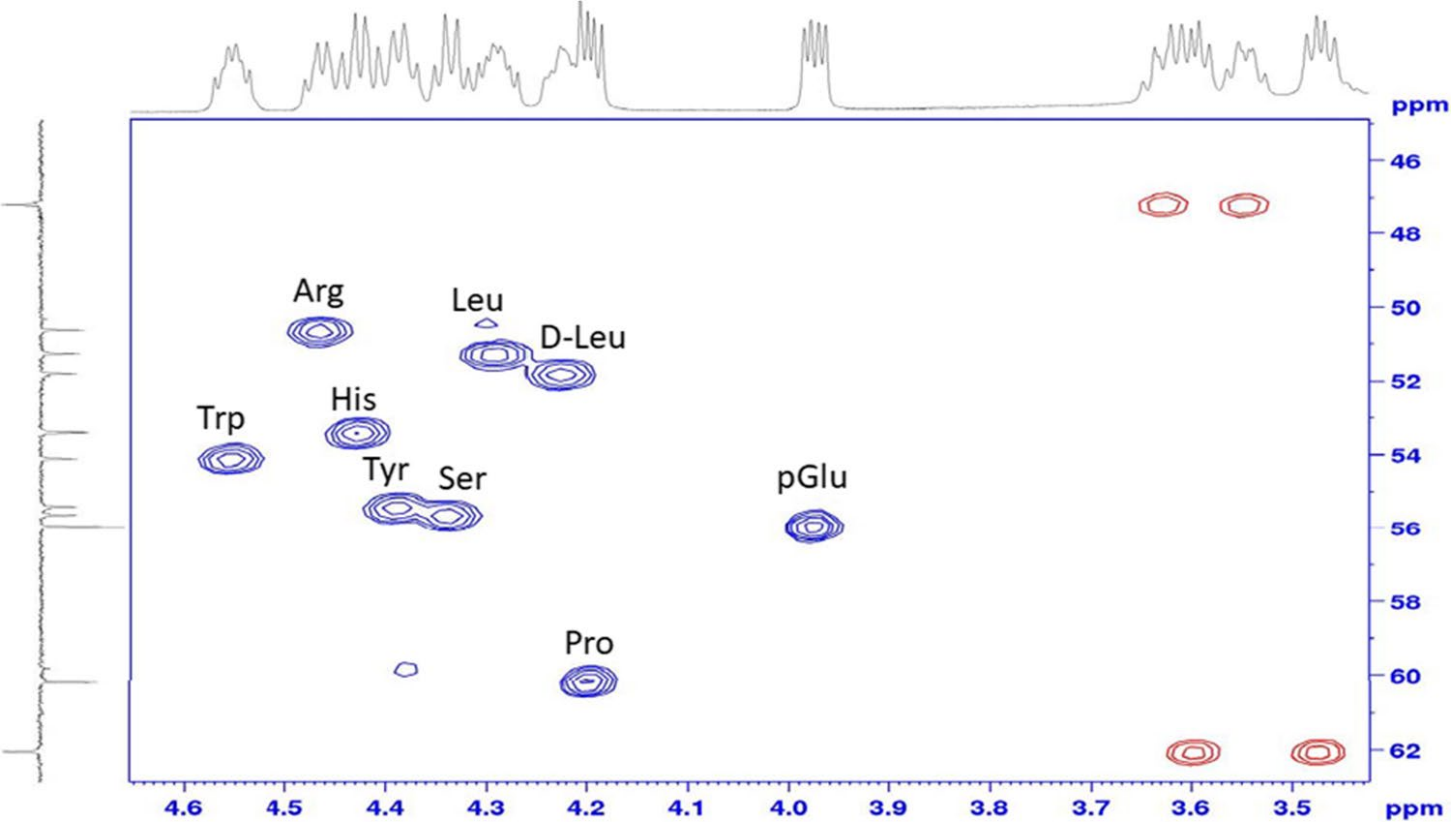
^1^H/^13^C HSQC NMR Spectrum for Leuprolide Acetate

NMR spectroscopy is a powerful tool for the determination of the chemical structure of a peptide. The proton, COSY, NOESY, TOCSY, carbon, ^1^H/^13^C HSQC, ^1^H/^13^C HMBC, ^1^H/^15^N HSQC, ^1^H/^15^N HMBC NMR spectra were collected for leuprolide acetate RS in Dimethyl Sulfoxide-d6 (+ 0.03% v/v TMS). The 1D and 2D NMR data are consistent with the structure of leuprolide acetate. The acquired proton resonances match to the reported literature values and the corresponding ^1^H-^1^H correlations obtained through ^1^H-^1^H COSY, ^1^H-^1^H TOCSY, and ^1^H-^1^H NOESY support the connectivity and all such information highly consistent with literature. Figure 1 shows an example of the ^1^H/^13^C HSQC NMR data used to confirm the identity of leuprolide acetate, demonstrating the connectivity of the alpha protons to alpha carbons in the respective amino acids.

#### Identity by Mass Spectrometry

**Fig. 2:**
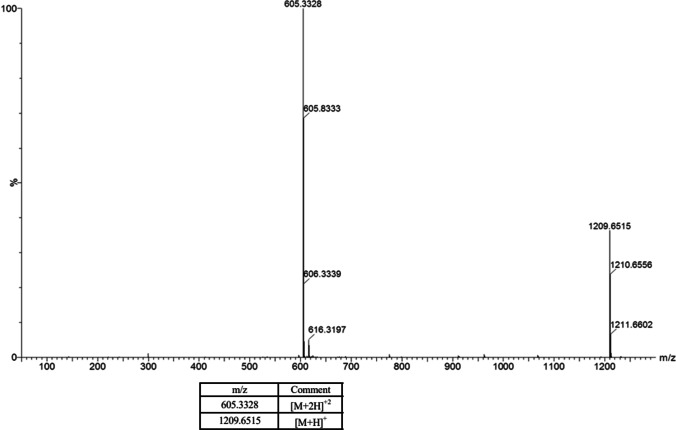
MS Spectrum of Leuprolide Acetate Bulk Material. The high-resolution MS spectrum for [M + H] and [M + 2H] leuprolide ions agrees with expected mass

**Fig. 3:**
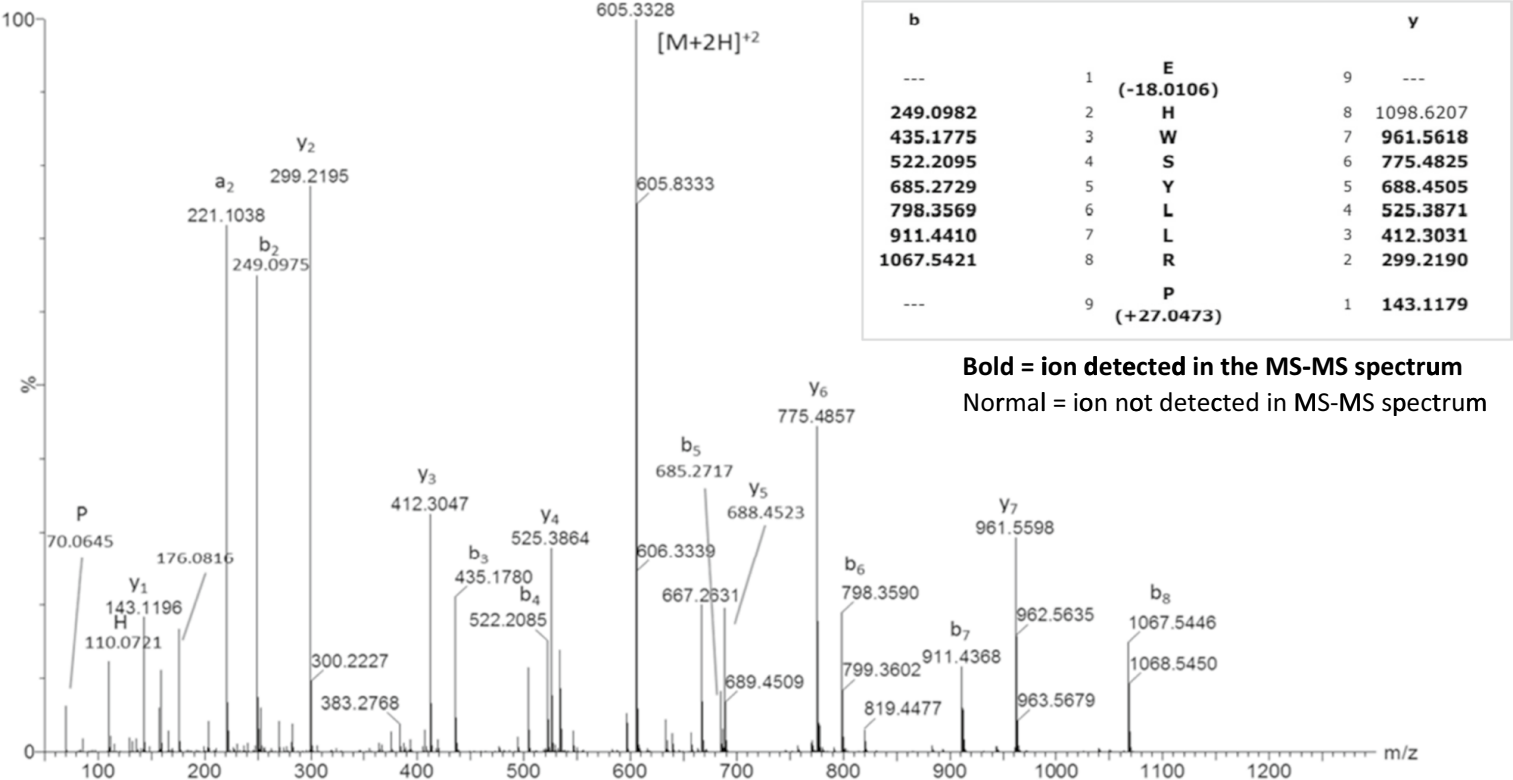
MS/MS Analysis of Leuprolide Acetate Bulk Material. The high-resolution MS/MS spectrum for [M + 2H] ion agrees with expected fragments for leuprolide

Mass spectrometry is often used to determine the monoisotopic mass of a peptide and to provide confirmation of the amino acid sequence using MS/MS fragmentation. For leuprolide, which has a theoretical m/z (monoisotopic, protonated) of 1209.6533, the experimental *m*/*z* was determined to be 1209.6515 which confirms the molecular mass of leuprolide (Fig. 2). In addition, MS/MS data yielded complete coverage of the amino acid sequence of leuprolide (Fig. 3).

#### Identity by HPLC-Retention Time

The HPLC-based identification test relies on the comparison of the retention times of the major peak from the test sample to an established reference standard. Co-injection of an equal mixture of the reference standard and the sample (identity sample solution) is often used in peptide monographs to confirm similar chromatographic performance. The acceptance criteria include that the retention time of the major peak of the sample solution is consistent with that of the standard solution, and the major peak of the identity sample solution and any co-injection elutes as a single peak. The bulk material and new lot were analyzed against the standard (current lot) per *Identification A, Leuprolide Acetate, USP-NF 2022*. The equivalent retention time of leuprolide peak in the standard and sample solutions were observed (Table IV), and a single peak was obtained in the co-injections, which included co-injection of the current lot with the bulk and new lot, as well as co-injection of the bulk and new lot. All these results met the acceptance criteria of the HPLC identification test in the USP leuprolide acetate monograph.

**Table IV:** HPLC Retention time of Leuprolide Acetate

Sample	Lab Test Results (retention time, minutes)				
	Lab L1	Lab L2	Lab L3	Lab L4	Lab L5
Current USP Lot	48.1	42.6	43.4	46.3	39.8
Bulk leuprolide acetate	48.2	42.6	43.2	46.5	39.3
Leuprolide acetate New Lot	48.4	42.6	43.5	46.5	39.5

#### Amino Acid Analysis (AAA)

AAA is used to determine the amino acid composition or content of peptides. Although AAA was commonly used as an identification test previously, it has been gradually replaced by other techniques, such as HPLC and MS. However, it is still a useful tool when discrimination between Leucine and Isoleucine is required. Bivalirudin is a single chain of 20 amino acids. The sequence is FPRPGGGGNG DFEEIPEEYL, which contains 1 leucine and 1 isoleucine. The amino acid content of bivalirudin was confirmed by amino acid analysis using capillary gas chromatography (Table V). The amino acid ratios of leucine and isoleucine are also consistent with the theoretical values, and further confirm the identity of bivalirudin.

**Table V:** Amino acid analysis of Bivalirudin

Amino Acids	Theoretical Amino Acid Ratio	Experimental Amino Acid Ratio
GLY	5	5.00
ILE	1	0.97
PRO	3	2.90
LEU	1	0.94
ASP	2	2.15
PHE	2	1.80
GLU	4	4.23
TYR	1	1.03
ARG	1	0.98

#### Chiral Testing

While most amino acids incorporated into peptides are L-nantiomers, some peptides also incorporate D-form amino acids. For example, bivalirudin contains a D-phenylalanine. Chiral analysis can be conducted to verify the presence of D-form in a peptide. The percent of amino acid in the D-form for bivalirudin was measured by capillary gas chromatography-MS on a chiral stationary phase. The GC–MS data indicated that approximately 50% of the total phenylalanine content was D-form (Table VI), which demonstrates the presence of D-form. Because the extent of racemization occurring during hydrolysis of the peptide required for the amino acid analysis cannot be estimated accurately, the percentage content of amino acids in D-form does not represent the enantiomeric purity of the peptide but can be used to establish the presence of the D-enantiomer.

**Table VI:** Content of amino acid in D-enantiomer conformation in Bivalirudin

Amino acid	[%] D-Amino acid
GLY	–
ILE	0.9
PRO	3.4
LEU	3.0
ASP	6.2
PHE	49.1
GLU	3.1
TYR	2.0
ARG	2.4

#### Value Assignment

USP employs a 2-step approach for the value assignment of USP’s peptide reference standards. First, the purity of the bulk material is determined. All detectable impurities (e.g., peptide related impurities, counter ion, water, residual solvents, non-combustible residues) are measured and subtracted from 100% to assign purity. Once the bulk purity is established, it is used as the standard to assay the peptide mass content of lyophilized vials (mg/vial) by the compendial HPLC assay method. To minimize the impact that differences in water content could have on the value assignment of the bulk, the moisture analysis is performed on three independent weighings (triplicate determinations) and all assays are performed on the same day. For results reported here, a mass balance approach was used for value assignment, but there are multiple methods for establishing peptide content, and approaches to value assignment continue to evolve as technologies advance. For example, Melanson and al. [11] reported a strategy where they corrected both LC–MS/MS and qNMR results for related peptide impurities using a Bayesian statistical approach using mass balance results as prior knowledge. In this study the authors used a peptide certified reference material. Our strategy of the development of peptides reference standards relies on the use of bulk materials that are reflective of the commercial Active Pharmaceuticals Ingredients (peptides API) and by using a mass balance approach, we found the least inter-laboratory variability when using a mass balance approach. Additionally, mass balance has been proposed as a benchmark for purity assessment of peptides [12].

#### Purity Determination of Bulk Material by Mass Balance

For mass balance purity assignment, all species other than the native peptide (e.g., impurities, counter ions) should be identified and measured. Table VII shows a theoretical example for a peptide with acetic acid as the counter ion and trifluoracetic acid as a reagent during synthesis. Residual solvents testing is used to account for any solvents from production that may still be present. Residue on ignition is used to account for any inorganic impurities. The determination of the number of laboratories selected to perform each test, the number of replicates, and the number of experiments is based on the methodology used and potential impact on the final calculation. The equation used to calculate purity separates HPLC impurities, which are measured as a % of the total detected area by chromatography, and counter ion / impurities measured on a weight/weight basis. Using this calculation, the theoretical example described in Table VII would have a purity of 0.93 mg of peptide (free base) per mg of material on the anhydrous basis.

**Table VII:** Testing results obtained for impurities/counter ions

Analyte/ *Reference*	Reported As	Average of n Lab results
Sum of HPLC impurities (I1)/*Monograph*	% Total Detected Area	0.92
Acetic acid Content (I2)/*USP* < *503* >	%w/w	5.58
Trifluoroacetic acid Content (I3)/*USP* < *503.1* >	%w/w	0.003
Residual solvents Content (I4)/*USP* < *467* >	%w/w	0.003
Residue on Ignition (I5)/*USP* < *281* >	%w/w	0.00

Purity = [(100.0—Σ%TDA) ÷ 100] × [(100.0 – Σ%w/w) ÷ 100]

= [(100.0 – (I1)) ÷ 100] × [(100.0 – (I2 + I3 + I4 + I5)) ÷ 100]

= [(100.0 – (0.92)) ÷ 100] × [(100.0 – (5.58 + 0.003 + 0.003 + 0.00)) ÷ 100]

= [(99.08) ÷ 100] × [(94.41) ÷ 100]

= [(0.99] × [0.94]

= 0.93 mg/mg

If water content was constant, it would be determined on a weight/weight basis like the other contributions above. However, in this example, the purity is further adjusted to the “as is” basis by applying the water content of the individual lab’s material. Water content from individual labs is used to account for differences in humidity during testing. Water content is determined at the time of preparation for performing the HPLC analysis to ensure that the water content is accurate (Table VIII).

**Table VIII:** Theoretical calculations are shown for L6 and L7

Lab	Purity (mg/mg)	%water	Water corrected purity (mg/mg)
L6	0.93	1.5	0.92
L7	0.93	2	0.91

^*^Note that more than two laboratories are required for assigning content

Once the purity of the bulk material is established, it is used as the physical standard to determine the peptide mass content of lyophilized vials (mg/vial) by a compendial HPLC analytical method. The purity of the final vialed material is also determined to ensure that no new impurities have been generated during the vialing process.

#### Peptide Mass Content Assignment for lyophilized Vialed Materials

Once the content of the bulk material has been established, it is used as a standard in the HPLC assay to define the content of the lyophilized vialed material. Bulk material is accurately weighed, solubilized, and used as the standard. The lyophilized vials are quantitively solubilized and used as the sample in the HPLC determination. The theoretical test results are given in Table IX. These results are corrected for the bulk material purity to obtain a final value for the content in the vials. If a current USP reference standard exists for the peptide, it is also used as an additional reference standard in the HPLC determination to assess consistency among the reference standards and samples.

**Table IX:** Peptide content of lyophilized vials assayed against bulk material

Vial Number	Lab L6	Lab L7
1	1.829	1.825
2	1.808	1.816
3	1.819	1.815
4	1.818	1.815
5	1.827	1.845
6	1.830	1.823
7	1.816	1.818
8	1.810	1.794
Average	1.820	1.819
%RSD	0.46%	0.78%

Vial content = [(Assay result L6 x corrected Purity value L6) + (Assay result L7 x corrected Purity value L7)]/n

= [1.820 × 0.92] + [1.819 × 0.91]/2

= [1.67 + 1.65]/2

= 3.32/2

= 1.66 mg/vial

#### Addressing Variability and Outliers

**Fig. 4:**
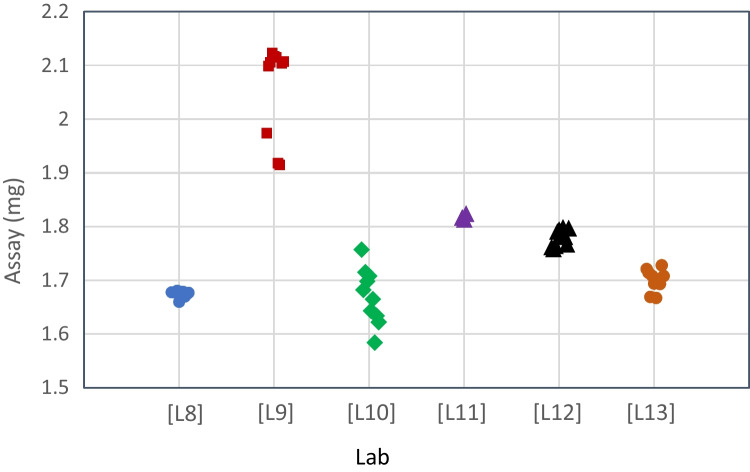
Variability of Value Assignment for Desmopressin Acetate Across Laboratories. Peptide mass content results for each of 10 preparations of the vialed material are shown for each laboratory to illustrate the variability both within and across laboratories (note that Lab L11 only assayed three preparations). Analysis of variance (ANOVA) showed that lab L9 results had statistically significant differences when compared to the mean of the other five labs

While some variability is usually observed across labs, it is important to identify outliers that can negatively impact the value assignment. Figure 4 shows an example of collaborative study results that showed high inter-laboratory variability and required use of a statistical approach to identify and eliminate outliers. Six collaborators participated in the evaluation. Each collaborator assayed ten sample preparations of lyophilized vials against the average of three standard preparations of bulk material. Analysis of variance (ANOVA) showed a statistically significant difference between labs (P < 0.0001), with Lab L9 being the outlier. Including the data from Lab L9 therefore has an undue influence on the overall value assignment. Furthermore, Lab L9 did not perform water content testing, so content for lab L9 was calculated using the average water content from the other five labs. Based on these issues, the data from Lab L9 was omitted from further analysis.

Tables X and XI illustrate the importance of the analysis and removal of outliers on the value assignment for the reference standard. Removal of the data from lab L9 reduced variability of the final value and resulted in narrower confidence intervals. The assay value of the material was defined based on the comparison to bulk and is 1.75 mg per vial on as is basis with a 95% confidence interval of 1.654 – 1.836. In addition to assigning the value against the bulk material, additional assays were conducted using the current reference standard lot and the European Pharmacopeia CRS to confirm the value assignment.

**Table X:** Value Assignment (All Labs)

Reference Lot	Mean	95% Confidence Interval
Bulk Material	1.783	1.629 – 1.938
Current Lot	1.691	1.595 – 1.786
Ph. Eur CRS	1.811	1.533 – 2.089

**Table XI:** Value Assignment (Lab L9 Removed)

Reference Lot	Mean	95% Confidence Interval
Bulk	1.745	1.654 – 1.836
Current Lot	1.727	1.660 – 1.795
Ph. Eur CRS	1.715	1.692 – 1.738

#### Stability

**Fig. 5:**
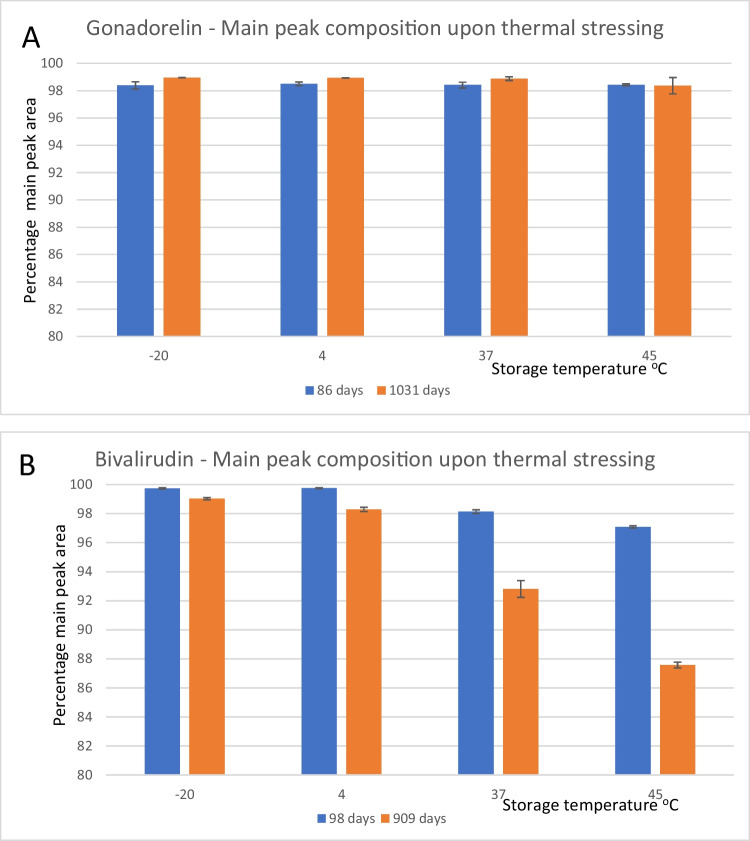
Accelerated Stability Studies of (**A**) Gonadorelin and (**B**) Bivalirudin. This figure shows the results of the accelerated stability testing where peptides were subjected to thermal stress at multiple temperatures. While gonadorelin was highly stable, bivalirudin was more susceptible to thermal stress and showed significant degradation at higher temperatures

Accelerated Thermal Degradation studies (ATD) were performed on trial fill materials and samples were analyzed using the HPLC impurity test in the corresponding USP monograph for each peptide. Frozen control samples were stored at -70°C and tested along with the various temperature conditions (-20°, 4°, 37°, and 45°C), at the timepoints shown. Evaluation of the stability of each peptide reference standard trial fill preparation was performed at elevated temperatures. Example results for gonadorelin and bivalirudin are shown in Fig. 5. Gonadorelin exhibited good stability at all temperatures and conditions tested, with a loss of less than 1% after 34 months at 45°C. In contrast, bivalirudin exhibited significant degradation at higher temperatures with losses of approximately 4% after 30 months at 4°C and 12% after 30 months at 45°C. Differences in stability of some of the peptide reference standards were observed under stressed conditions at higher temperatures. While most peptide reference standards are stored at -20°C or colder, ATD studies can be used to inform shipping conditions for the final lyophilized product and frequency of real-time stability testing.

The final labeled USP reference standards are placed on a real-time stability monitoring program, called Continued Suitability for Use (CSU). Reference standards are stored at the prescribed conditions (2–8 °C, -20° C or colder, etc.) and removed at designated intervals to assess purity and potency. The CSU data is trended, and materials that are out of specification or are trending towards being out of specification using established alert and action limits will be scheduled for replacement or quarantined and replaced.

## Conclusion

Reference standards are critical components of the control strategy for therapeutic peptides, as they support the test methods used to assess the critical quality attributes for these important pharmaceuticals. Because a reference standard is the physical material to support testing of the quality of a drug substance and drug product, the reference standard requires a level of characterization beyond the compendial tests for which the reference standard is used. This enhanced characterization often requires multiple orthogonal techniques to confidently understand, verify, and quantify the characteristics of the reference standard. For example, multi-laboratory studies used for characterization of reference standard candidates typically use 1) multiple orthogonal identification methods such as NMR, peptide mapping, HPLC, and amino acid analysis to establish identity and evaluate misincorporation and other modifications, 2) multiple methods to evaluate candidate reference standards for impurities and 3) mass balance-based value assignments to ensure that all elements (e.g., counterions, residual solvents) have been considered in the value assignment.

Variability or bias in the methods used for value assignment or changes in the reference standard upon storage can result in inaccurate assignment of content and lead to incorrect dosing. The multi-laboratory testing approach used by pharmacopeias and standard setting organizations, such as USP, captures the analytical variability and addresses potential laboratory bias in the reference standard assigned values. The data collected from multiple laboratories is analyzed as a whole, using appropriate statistical approaches to identify and eliminate outliers. To address stability concerns, the formulation and configuration of peptide reference standards are designed to support long term storage and accelerated stability studies are conducted to model potential degradation over time. In addition, their stability is monitored in real time to confirm they are valid for use while publicly available in a catalog. In summary, this report summarizes the scientific rigor and best practices used to establish compendial reference standards that play a key role in supporting the quality of medicines people rely on. It is worth noting that if these reference standards are used for applications other than those for which it was developed and specified, additional characterization may be required.

## Data Availability

The data presented in this publication are available from the corresponding author on reasonable request.

## Declarations

### Disclaimer

Certain commercial equipment, instruments, or materials are identified in this article to adequately specify the experimental procedure. Such identification does not imply approval, endorsement, or certification of a particular brand or product by the authors and their employers; nor does it imply that the equipment, instrument, or material is necessarily the best available for the purpose, or that any other brand or product was judged to be unsatisfactory or inadequate.
